# Nestling Growth and Brood Reduction in the Southern Yellow‐Billed Hornbill ( *Tockus leucomelas* )

**DOI:** 10.1002/ece3.72147

**Published:** 2025-12-09

**Authors:** Melanie A. Smith, H. Luke Anderson, Jordan Karubian, Mark Stanback

**Affiliations:** ^1^ Tulane University New Orleans Louisiana USA; ^2^ Syracuse University Syracuse New York USA; ^3^ Davidson College Davidson North Carolina USA

**Keywords:** brood reduction, cavity nesting, hatching asynchrony, hornbills, nestling growth, nestling mortality, *Tockus leucomelas*

## Abstract

Hatching asynchrony is common among altricial bird species and has long been hypothesized to facilitate brood reduction, which in turn may maximize reproductive outputs in environments with variable resource availability. Despite its prevalence, the drivers and mechanisms of brood reduction are not well understood for many species. The southern yellow‐billed hornbill, *Tockus leucomelas* , is a useful model for understanding brood reduction because it exhibits extreme hatching asynchrony and a unique nesting strategy where the female and the brood are sealed within a cavity, sheltered from predators. Here, we documented aspects of breeding biology in artificial nest boxes and assessed the influence of various factors on nestling growth and mortality. Earlier‐hatched nestlings had higher growth rates throughout development and were more likely to survive to fledge than their younger siblings. Maternal presence in the nest had positive impacts on growth rates of later‐hatched nestlings, likely reflecting the female's role in mitigating sibling competition. Despite differences in growth and survival during the nesting period, weight at fledging did not differ according to hatch order, likely because later‐hatched nestlings spend more time in the nest before fledging. Using a path analysis, we show that brood size and growth rates of youngest nestlings are significant direct predictors of brood reduction. In addition, our analysis suggests that rainfall (a proxy for resource availability) may indirectly influence the likelihood of brood reduction via effects on growth rate of the youngest nestling, although estimated effect sizes were small. The observed relationships between hatch order, brood size, nestling growth and mortality, and environmental variables provide support for some predictions of the brood reduction hypothesis for the function of hatching asynchrony and advance our understanding of brood reduction dynamics in this species.

## Graphical abstract

In the southern yellow‐billed hornbill, *Tockus leucomelas* , we found first‐hatched nestlings had a higher growth rate and that growth rate was negatively correlated with mortality, such that early hatching nestlings were more likely to survive than their younger siblings. Brood reduction was more likely to occur in larger broods and was predicted by the growth rate of the youngest nestling. However, weight at fledging did not differ between hatch orders, indicating that the southern yellow‐billed hornbill fledglings may face limited impacts of hatching order if they survive brood reduction.

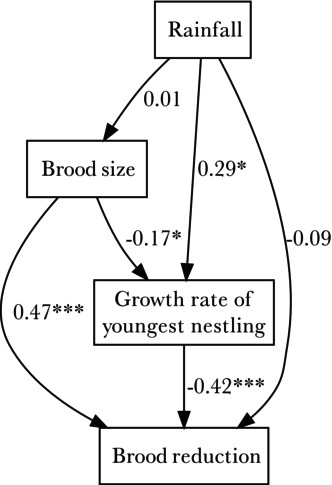

## 1 Introduction

Hatching asynchrony—where parents begin incubating eggs before the entire clutch is laid, resulting in staggered hatching dates and a brood consisting of nestlings at different developmental stages (Stenning 1996; Viñuela 2000)—is a common phenomenon in altricial bird species. At first glance, hatching asynchrony appears paradoxical, given the potentially negative fitness consequences of disadvantaging later‐hatched offspring (Forbes 2011; Hildebrandt and Schaub 2018; Mainwaring et al. 2009). Thus, considerable theoretical, observational, and experimental effort has been dedicated to explaining the evolutionary basis of hatching asynchrony over the past century (reviewed in Stenning 1996; Węgrzyn et al. 2023). Hypotheses for its evolution and maintenance, ranging from adaptive to incidental (Węgrzyn et al. 2023), include: providing insurance against unviable eggs, predation events, or developmentally poor offspring (Forbes et al. 2002; Morandini and Ferrer 2015; Stinson 1979); reducing sibling rivalry or parental foraging demands (Lee and Lima 2017; Stenning 1996; Stinson 1979); and arising as a byproduct of pressure to begin incubation early to avoid nest predation (Clark and Wilson 1981; Hussell 1972) or brood parasitism (Stoleson and Beissinger 1995).

Among the longest‐standing and most influential hypotheses for the adaptive significance of hatching asynchrony is that it serves to facilitate brood reduction, or the strategic winnowing of the brood size to the maximum number of offspring a breeding pair can support (Lack 1947; Lee and Chung 2020; Morandini and Ferrer 2015; Pijanowski 1992). In asynchronously laid clutches, later‐hatched “marginal” nestlings are developmentally behind earlier‐hatched ones, and thus they can easily be sacrificed in favor of the “core” brood if necessary (Forbes 2010). Such facultative brood reduction is generally thought to be tied to resource availability (Lack 1947; Pijanowski 1992): when resources are scarce and parents are unable to feed all nestlings, parents may improve their fitness by favoring some offspring over others, typically the larger and stronger sibling(s) (Clark and Wilson 1981; Lee and Chung 2020). Work in yellow‐headed blackbirds ( *Xanthocephalus xanthocephalus* ) has shown that hatching asynchrony and associated brood reduction was advantageous in resource‐poor years, while minimally costly in resource‐rich years (Forbes et al. 2002). In addition, studies in magpies ( *Pica pica* ) have demonstrated that experimental increases in clutch size resulted in nestlings with poorer body condition than in control nests, suggesting that brood reduction may allow parental birds to dynamically adjust clutch size to the maximum number of offspring that can be supported given available resources (Husby 1986). Thus, during periods of resource unpredictability, the combination of hatching asynchrony and brood reduction may increase the likelihood that at least some nestlings will receive enough resources to survive (Howe 1978; Stenning 1996).

Brood reduction can be achieved via diverse mechanisms. Typically, brood reduction is effectuated via mortality of the smallest, youngest nestling(s) through starvation, competition, fratricide, or infanticide (Husby 1986; Lee and Chung 2020; Morandini and Ferrer 2015; Forbes et al. 2002; Hildebrandt and Schaub 2018; Wesołowski 2023). However, parental strategies vary within and across species, with some favoring the survival of younger nestlings (Shizuka and Lyon 2013) and others only reducing food allocation for last‐hatched nestlings of a particular sex (McDonald et al. 2005). More extreme examples of parental influence during brood reduction involve infanticide and/or cannibalism of the youngest nestling by the mother, where the dead nestling is either consumed by the mother or fed to other nestlings (Chan et al. 2007; Finnie 2012; José Soler et al. 2022). Even within a species, parental strategies can vary. In southern yellow‐billed hornbills ( *Tockus leucomelas* ), mothers have been documented both mitigating sibling competition to benefit the youngest nestlings, and cannibalizing the youngest nestlings (Finnie 2012). The presence of these two conflicting strategies within the same species suggests that parental approaches to managing brood size are context‐dependent, likely influenced by environmental conditions, food provisioning, offspring age, and offspring condition. Documenting and understanding the variation in brood reduction dynamics within and across species is therefore an important goal, given the crucial role it may play in enabling parents to successfully raise offspring in unpredictable environments.

Hornbills (Aves: Bucerotidae), a diverse family of cavity‐nesting birds occurring in tropical and subtropical regions of Asia and Africa, provide a particularly interesting opportunity to study the relationship between hatching asynchrony and brood reduction. African hornbill eggs are known to hatch as many as 4–7 days apart (Combrink et al. 2020; Stanback and Engelbrecht 2024), resulting in dramatic size and developmental differences between the oldest and youngest nestlings. In an extreme example, Monteiro's hornbills ( *Tockus monteiri* ) may exhibit up to 14‐day age differences between the oldest and youngest siblings in a nest (Kemp and Kemp 1972; Stanback and Engelbrecht 2024), among the largest hatching asynchrony recorded in birds. These differences in age and size between hornbill siblings may play an important role in facilitating brood reduction, given that later‐hatched nestlings would be significantly weaker competitors compared to earlier‐hatched, larger siblings, and many African hornbills live in arid environments with unpredictable resources, where brood reduction is likely to be beneficial.

**FIGURE 1:**
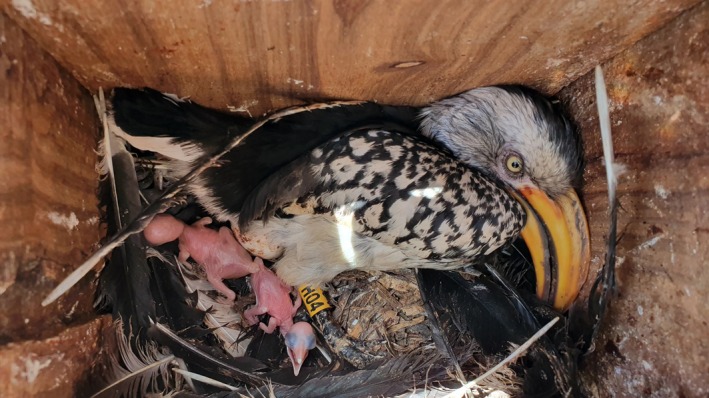
Inside a southern yellow‐billed hornbill nesting cavity. Photograph depicts a female southern yellow‐billed hornbill ( *Tockus leucomelas* ) in a nest box with her two nestlings. Photo credit: Dirk Heinrich.

This study uses a multi‐year dataset to characterize the factors shaping nestling growth and brood reduction in a Namibian population of southern yellow‐billed hornbills ( *Tockus leucomelas* ; Figure 1). Our first major aim was to assess the potential environmental and demographic factors influencing nestling growth rates, including resource availability (expected to positively predict nestling growth rates), maternal presence in the nest (expected to mediate sibling competition), and hatch order (expected to be associated with relative nestling size and competitive ability). In line with the hypothesis that hatching asynchrony functions to enable brood reduction, we predicted that nestling growth and survival would be significantly influenced by hatch order (with later‐hatched siblings exhibiting lower growth rates and higher mortality than earlier‐hatched siblings) and resource availability.

Our second major aim was to assess the degree to which various factors—including resource availability, brood size, and growth rates of the youngest nestling—predicted the incidence of brood reduction in this species. If brood reduction serves as a bet‐hedging strategy in unpredictable environments, we predicted that it would be more likely to occur in years with low resource availability. Our proxy for resource availability was annual rainfall, which is a major determinant of vegetation and insect availability in arid environments such as Namibia (Lehmann et al. 2020). In addition, we hypothesized that brood reduction would be more likely to occur in larger broods, given that these broods have more potentially expendable, “marginal” offspring. Lastly, from a proximate perspective, we hypothesized that brood reduction is likely achieved via starvation of the youngest nestling, as has been observed in other species (Boland et al. 1997; Braun and Hunt 1983; Clark and Wilson 1981; Lee and Chung 2020); thus, we expected that growth rate of the marginal nestling would negatively predict the occurrence of brood reduction in a nest. Given that these various predictors of brood reduction may also causally influence one another (e.g., brood size may influence growth rate of the youngest nestling), we fit a path model to assess support for a variety of direct and indirect causal pathways by which the environment could influence brood reduction and its predictors. Altogether, this study aims to shed light on the function of hatching asynchrony, the mechanisms of brood reduction, and the effects of various ecological and demographic factors on nestling growth and survival.

## 2 Methods

### 2.1 Study System

Hornbills exhibit an unusual enclosed nesting strategy: the female seals herself inside a cavity, lays her eggs, molts her feathers, and relies on the male for food until she has regrown her feathers, at which time she emerges from the nesting cavity and the largest nestling reseals the hole (Figure 1; Kemp 1969; Mills et al. 2005; Stanback and Engelbrecht 2024). This unique cavity‐nesting behavior makes hornbills well‐suited for studying the phenomenon of brood reduction, as it largely removes the potential for predation events to confound nestling mortality data (Brightsmith 2005). The number of eggs laid varies by species, but *Tockus* hornbills typically lay 2 to 6 eggs (Stanback and Engelbrecht 2024). While the female is in the nest, she can allocate food among the nestlings by feeding bill‐to‐bill, thereby mediating sibling competition and encouraging the survival of younger nestlings (Finnie 2012). However when she leaves the nest, the nestlings compete directly for the food provided by the parents (Finnie 2012). Nestlings of the southern yellow‐billed hornbill ( *Tockus leucomelas* ), our study species, have been shown to exhibit reduced sibling‐sibling aggression and lower disparities in resource acquisition when the mother is present (Finnie 2012).

As long‐lived species, female African hornbills are expected to prioritize their own survival over that of their offspring, making future reproductive efforts more important than the current one (Mills et al. 2005). Thus, southern yellow‐billed hornbill mothers often leave the nesting cavity before the youngest nestling is able to eat on its own or compete effectively with its older siblings for food. Several studies have found dying or dead nestlings in nests, and nestlings have even disappeared altogether (Chan et al. 2007; Engelbrecht 2013; M. T. Stanback pers. obs.). One female was documented on a nest cam killing a nestling and subsequently using its corpse to feed its siblings (Engelbrecht 2013), and another study documented females consuming both eggs and nestlings (Finnie 2012). Yet beyond these observations, brood reduction dynamics remain poorly documented for most hornbill species, and the importance of sibling competition and female mediation (or lack thereof) remains poorly understood.

### 2.2 Data Collection

Field work was conducted during the austral summer on the Daan Viljoen Game Reserve (DVGR) near Windhoek, Namibia (1984; 1995–1996), and during austral spring and/or summer on land owned by the Cheetah Conservation Fund (CCF) near Waterberg Plateau National Park (2017–2020; 2022–2023). We collected data over 8 different breeding periods: January–March 1984; April 1995; March–April 1996, February–May 2017; December 2017–February 2018; January–April 2019; December 2019–March 2020; and November 2022–January 2023. Southern yellow‐billed hornbills have a bimodal breeding season, with some females breeding in both spring and summer months (Brown et al. 2014; Stanback et al. 2021). There is no documented difference in nesting behavior between the two seasons. Duration of data collection in a given seasons was determined by natural variation and logistics of our field campaigns, and sampling months were designed to encompass as much of breeding seasons as logistically possible.

While hornbills naturally nest in tree cavities, pairs will also breed in nest boxes attached to tree trunks (Riekert and Clinning 1985), and our results are based on southern yellow‐billed hornbill pairs that chose to breed in the nest boxes that we attached to trees (*n* = 125 total nests). There is no evidence that nesting patterns differ between artificial nest boxes and natural conditions. Nest boxes in this study were made from either plastic, wood planks, or plywood, contained entrance holes 60 mm in diameter, and were installed about 1.5 m above the ground.

Nest boxes were checked roughly every 2–3 days. As observed in natural tree cavities, females sealed themselves into the nest boxes; once the female left the nest, the nestlings, typically the oldest, resealed the nest box. Our nest boxes had a doorway that was used to access and examine the brood. During each check, we measured the body mass of any hatched nestlings to the nearest gram using a bag and spring scale. We also recorded female presence (i.e., whether the mother was in the nest box) and brood size (i.e., the number of nestlings in the nest box). Nestlings were eventually banded once the female left the nest, but individuals were also easily identifiable prior to banding due to size discrepancies resulting from extreme hatching asynchrony. We also inferred hatching dates (exact dates were unknown because boxes were not checked daily), tracked brood size, and noted instances of nestling mortality, fledging, or disappearance.

Brood reduction was assumed to have occurred in cases where nestlings disappeared prior to reaching fledging age; nestlings disappearing after fledging age were considered to have fledged. Our cutoff for minimum age at fledging was 40 days post‐hatching (Kemp and Boesman 2020), which was conservative to minimize instances where successful fledges were misattributed as cases of brood reduction. Notably, many nestlings that disappeared before fledging age were observed to have been starving and losing mass prior to their disappearance. Alternative explanations for nestling disappearance besides brood reduction include predation, illness, or congenital diseases. While we cannot definitively rule out the latter two explanations in any cases of putative brood reduction, predation events were obvious and rare: in the few cases where predation did occur, predators (usually a honey badger, *Mellivora capensis* ) would tear open the nest plug, typically resulting in total nest mortality. Any observations from a nest after a predation event were excluded from analyses, and mortality from predation was not classified as brood reduction. 

Local annual precipitation data for all field seasons was obtained from Climate Hazards Group InfraRed Precipitation with Station (CHIRPS) (Funk et al. 2015). We chose to use annual rainfall rather than spring rainfall given that nesting took place during both spring and summer, and time lags between rainfall and resource availability are unknown. We acknowledge the limitation of using annual precipitation as a proxy for resource availability, but higher‐resolution prey abundance metrics were unavailable.

### 2.3 Growth Curve Analyses

**FIGURE A1:**
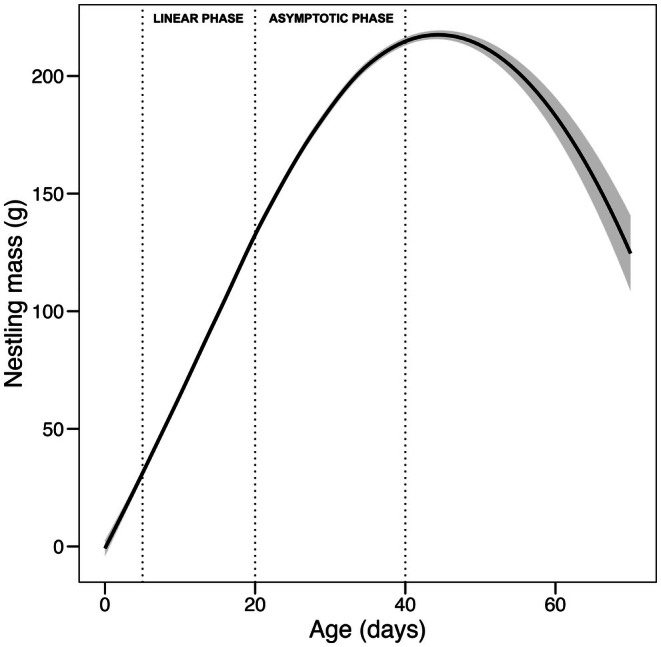
Southern yellow‐billed hornbill nestling growth trajectories across development. The bounds of the linear (6–19 days) and asymptotic (20–39 days) growth phases are shown with dashed lines.

In vertebrates, mass change as a function of age is typically sigmoidal (Erickson and Tumanova 2000; Sussman 1964), and thus growth can be divided into exponential, linear, and asymptotic phases (Myhrvold 2013). This variation in growth rate across development needed to be accounted for when assessing the effect of different factors of interest (e.g., hatch order, female presence) on nestling growth rates. For instance, the growth rates of an older nestling in the asymptotic phase of its growth curve might be lower than the growth rate of the youngest nestling still in the linear phase of its growth curve, and averaging across the full trajectory might obscure the effects of various factors on growth within a particular phase, particularly if the frequency or timing of measurement was inconsistent across phases or individuals. Thus, to standardize growth rate comparisons between nestlings of different hatch orders, we determined the age at which nestlings typically enter each phase of the growth curve. To do this, we first plotted the growth trajectories of all hornbill nestlings in the dataset to generate an overall mean growth curve (Figure A1). We then delineated the three phases of this growth curve (exponential, linear, and asymptotic) by visually examining the points at which the curve departed from parallel with a reference line (i.e., deviations from a constant slope). The exponential phase was defined as the period of accelerating growth prior to the point at which the mean growth curve exhibited a constant slope; the linear phase was defined as the period when the mean growth curve exhibited a constant slope; and the asymptotic phase was defined as the period bounded by the inflection point in the mean growth curve (i.e., the point where the slope began to decelerate and deviate from constant slope) and the earliest potential fledging timepoint (40 days post‐hatching). We were then able to standardize analyses by comparing only growth rates within the same growth phase. We limited our analysis of nestling growth rates to the linear and asymptotic phases, as our nest visits were apparently not frequent enough to reliably capture the initial burst of growth during the exponential phase (presumed to occur sometime between Days 0 and 5; Figure A1).

### 2.4 Statistical Analyses

All analyses were performed using RStudio version 2024.4.2.764 (Posit Software 2024). For descriptive information about aspects of breeding biology, we present descriptive statistics in the form of means ±1 standard error of the mean (SEM). We then sought to analyze the different factors influencing nestling growth and incidence of brood reduction using several linear and generalized linear mixed models (outlined below). Because sample sizes were low for fourth‐ and fifth‐hatched nestlings (*n* = 5 and *n* = 2, respectively, during the linear phase), they were excluded from all growth analyses described below; however, these nestlings were considered in analyses related to brood reduction.

To assess the degree to which hatch order predicted a nestling's residual mass at a given age, we fit a robust linear mixed‐effects model (to account for outliers and heavy tails) using the *rlmer* package in R (Koller 2016). The model included nestling residual mass (extracted from the second‐order polynomial regression of mass vs. age) as a function of hatch order, as well as a hierarchical random effect of nestling ID within nest ID. We then conducted a Tukey post hoc test using the *emmeans* package (Lenth 2023) to obtain pairwise comparisons between hatch order classes. We ran two versions of this model: one considering only the linear phase of nestling growth (i.e., between 6‐ and 19‐days post‐hatching) and another considering only the asymptotic phase (i.e., between 20‐ and 39‐days post‐hatching). Deviations from linearity during the asymptotic phase were relatively mild, and thus growth trajectories in this phase were still reasonably approximated using a linear model. In addition, we assessed whether age and/or mass at fledging differed according to hatch order using ANOVAs and linear mixed‐effects models, the latter of which included nest ID as a random effect.

Next, we aimed to assess the relative influence of various factors on nestling growth during the linear growth phase. The constant growth expectation during the linear phase enabled us to estimate the effects of these factors on individual growth rates; in contrast, the effects of various factors during the asymptotic phase would be difficult to disentangle from expected gradual declines in growth rates, particularly given random variation in the exact timepoints along the growth curve that individuals were measured. We again fit a robust linear mixed‐effects model (to account for outlier presence) using the *rlmer* package (Koller 2016), with individual growth rates during the linear phase modeled as a function of hatch order, female presence (i.e., whether the mother was present in the nest box or had departed), and total annual rainfall (mm). Nestling ID within nest ID was again included as a hierarchical random effect.

We also aimed to assess the impact of nestling hatch order on age at death in cases of brood reduction. We fit a linear mixed‐effects model in *nlme* (Pinheiro et al. 2017), with age at death (square root‐transformed to increase the normality of residuals) modeled as a function of hatching order. Qualitative results were similar when the dependent variable was untransformed. Because brood reduction occurred more than once in some nests, nest ID was included as a random effect in this model.

Finally, we aimed to integrate potential predictors of brood reduction (brood size, growth rate of youngest nestling, and rainfall) into a single conceptual and analytical framework. To do this, we fit a path model in *lavaan* (Rosseel 2012) that represented our hypotheses for how key variables could directly and indirectly influence the incidence of brood reduction, which was coded as a binary response variable. As in other forms of causal modeling, path models represent hypothesized chains of causation between variables of interest, with single‐headed arrows representing the direction of causation. Intercorrelations between variables can then lend support for various direct and indirect causal pathways (but cannot definitively prove causation). We hypothesized that the environment may directly influence brood size (if females are able to lay more viable eggs in resource‐rich years), growth rates of marginal nestlings (if pairs are able to better support all nestlings in the brood in resource‐rich years), and brood reduction (if females directly assess the environment to decide whether to eliminate certain nestlings). In addition, we hypothesized that mediating variables such as brood size (which may influence the strength of sibling competition and the number of “expendable” nestlings) and growth rate of the youngest nestling (which should reflect starvation) may be causal pathways by which rainfall influences the probability of brood reduction. In some nests (*n* = 36), the youngest nestling perished before multiple measurements could be taken, and thus growth rate calculations were impossible; in these cases, we used the growth rate of the second‐youngest nestling in the brood for analyses. Only nests for which data were available for all variables of interest were retained in the analysis (*n* = 96 nests total; *n* = 60 and *n* = 36 nests with and without brood reduction, respectively). We note that because the hypothesized model was just‐identified (i.e., 0 degrees of freedom), we were unable to calculate fit indices for the model. In addition, due to the presence of an outlier growth rate observation (> 4 SD from the mean) that altered path significance, we ran two versions of the model—one with and one without the outlier. The version of the model excluding the outlier is presented in the main text, while the version including the outlier is reported in the appendix.

## 3 Results

### 3.1 Descriptive Breeding Information

**FIGURE A2:**
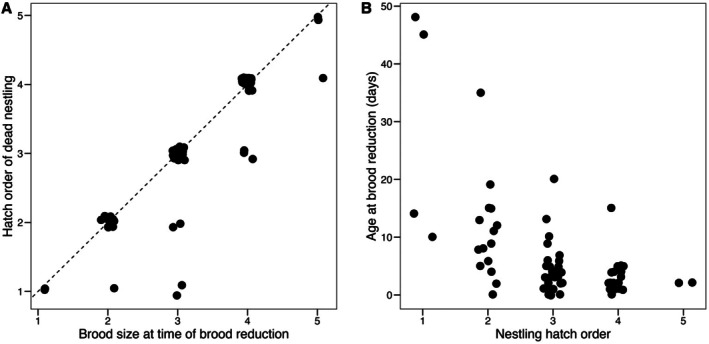
Impact of hatch order on brood reduction probability and age at mortality. (A) In 88.6% of known cases of brood reduction, the youngest nestling in the brood died. Such instances are represented by points that fall along the dotted line (i.e., where the hatch order of the dead nestling is equal to the size of the brood). (B) Hatch order significantly predicted the age at which brood reduction occurred (LMM: *t* = −6.60, df = 19, *p* < 0.0001), with later‐hatched nestlings tending to die at earlier ages than earlier‐hatched nestlings.

We recorded data on 353 nestlings in 125 nests. The average brood size was 2.88 nestlings (± 0.09; range 1–5). Out of the 125 nests, 48% experienced brood reduction (*n* = 60) and 30% did not (*n* = 37). We were unable to confidently conclude whether brood reduction occurred or if nestlings successfully fledged in 22% of nests (*n* = 28), and thus those nests were excluded from analysis. Nests where brood reduction occurred had significantly larger broods than those in which brood reduction did not occur (3.28 ± 0.10 vs. 2.35 ± 0.17 nestlings; Wilcoxon test: *W* = 1720, *p* < 0.001). In nests where brood reduction occurred, we were able to confidently identify which nestling(s) died in 76 instances. In 88.2% of these cases, the youngest nestling in the brood perished (Figure A2A). On average, nestlings died via brood reduction at 6.63 days (±1.06) post‐hatching, although we observed a wide range between 0 and 48 days. The timing of mortality via brood reduction was dependent on the nestling's hatch order, with later‐hatched nestlings dying at significantly younger ages than earlier‐hatched nestlings (LMM: *t* = −6.60, df = 19, *p* < 0.0001; Figure A2B).

### 3.2 Effects of Hatch Order on Growth Rates and Weight at Fledging

**FIGURE 2:**
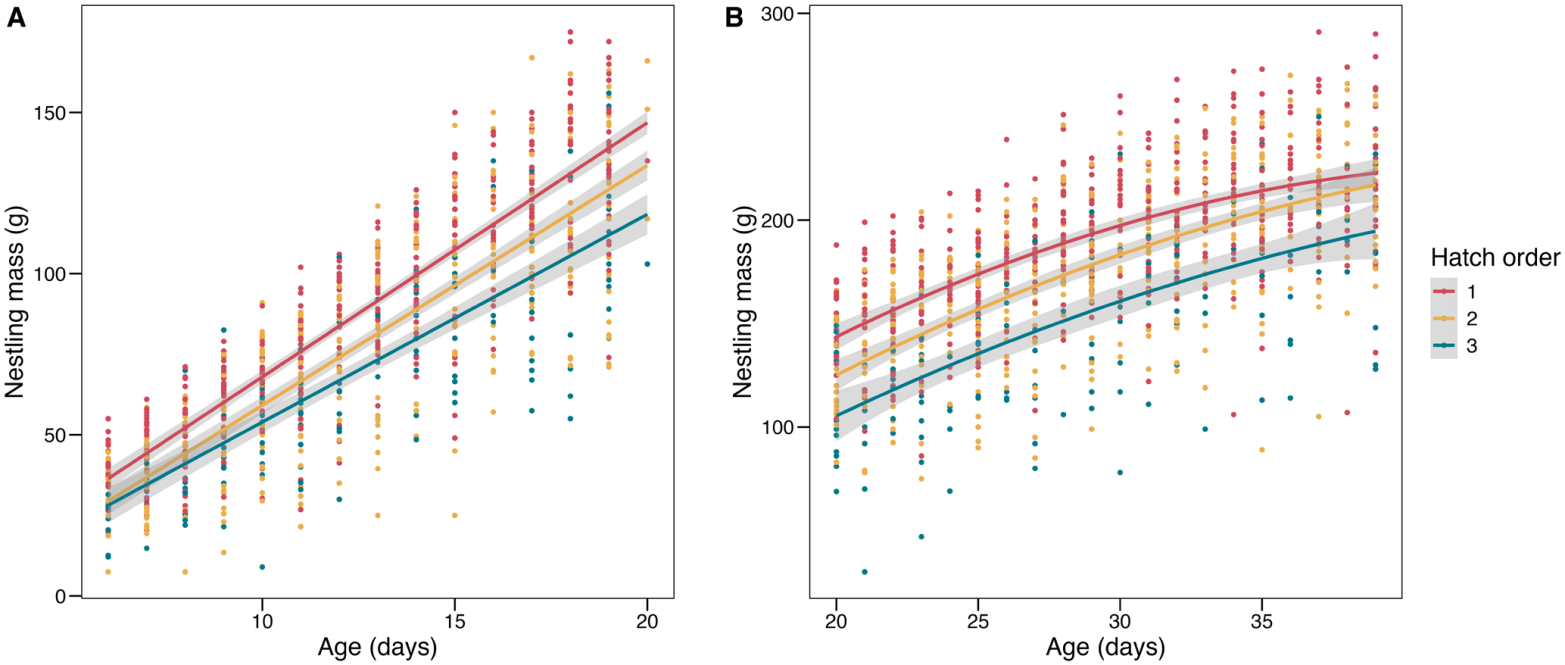
Hatch order influences nestling mass during development. Nestling mass at a given age significantly differed by hatch order during (A) the linear growth phase (Days 6–19 post‐hatching) and (B) the asymptotic growth phase (Days 20–39 post‐hatching). Shaded areas represent 95% confidence intervals.

Throughout the linear growth phase, earlier‐hatched nestlings were significantly heavier than their younger siblings (Figure 2A): nestlings that hatched first (*n* = 89) were heavier on average than second‐hatched nestlings (*n* = 83; *t* = 4.00, df = 124, *p* < 0.001) and third‐hatched nestlings (*n* = 44; *t* = 5.87; df = 124; *p* < 0.001), and second‐hatched nestlings were heavier than third‐hatched nestlings (*t* = 2.55; df = 124, *p* = 0.03). Differences between nestlings of different hatch orders persisted during the asymptotic growth phase (Figure 2B): first‐hatched nestlings (*n* = 84) were significantly heavier at a given age than second‐hatched nestlings (*n* = 70; *t* = 2.63, df = 99, *p* = 0.03) and third‐hatched nestlings (*n* = 33; *t* = 5.77, df = 99, *p* < 0.001), and second‐hatched nestlings were heavier than third‐hatched nestlings (*t* = 3.61, df = 99, *p* < 0.001). Note the reduction in sample size between growth phases are due to nestling mortality.

**FIGURE 3:**
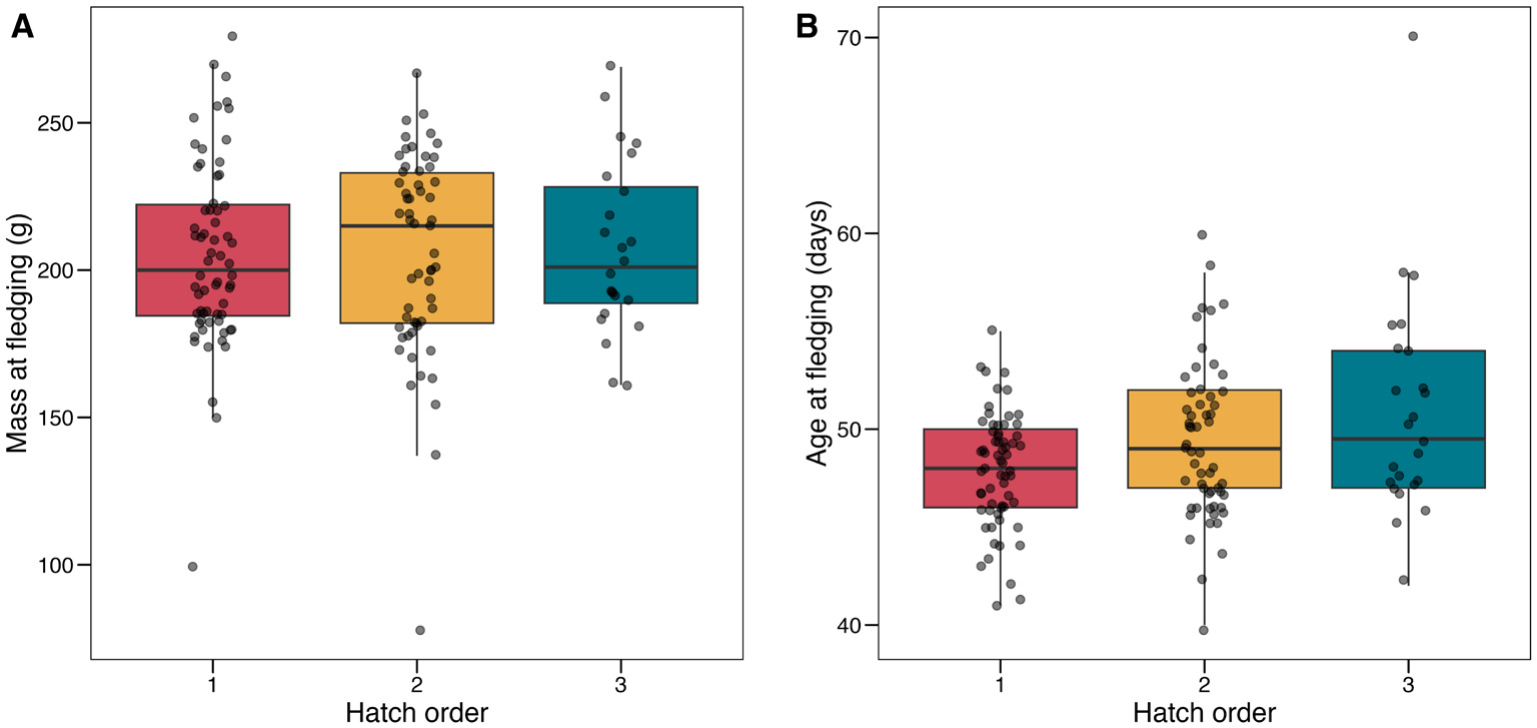
Mass at fledging does not differ among hatch orders, likely because later‐hatched nestlings spent more time in the nest prior to fledging. (A) Nestling mass at fledging did not significantly differ based on hatch order (p > 0.99 for all post‐hoc pairwise comparisons). (B) Age at fledging significantly differed across hatch orders, with first‐hatched nestlings fledging significantly earlier than second‐hatched (*t* = −2.07, df = 76, *p* = 0.04) and third‐hatched nestlings (*t* = −3.93, df = 76, *p* < 0.01).

Despite differences throughout the entire nesting period, weight at the time of fledging did not differ by hatch order among nestlings that survived to fledge (ANOVA: *F*_2,142_ = 0.03, *p* = 0.97; Figure 3A; Table 1). This may be because second‐hatched (LMM: *t* = 2.07, df = 76, *p* = 0.04) and third‐hatched nestlings (*t* = 3.93, df = 76, *p* < 0.001) remained in the nest for significantly longer than first‐hatched nestlings (Figure 3B; Table 1). Based on a post‐hoc Tukey’s test, third‐hatched nestlings remained in the nest significantly longer than both first‐hatched (*t* = −3.23, df = 76, *p* < 0.001) and second‐hatched nestlings (*t* = −2.40, df = 76, *p* < 0.05), although there was not a pairwise difference between first‐ and second‐hatched nestlings (*t* = −2.07, df = 76, *p* = 0.10).

**TABLE 1:** Descriptive statistics of nestling age and mass at fledging by hatch order.

Hatch order	Mean age at fledging (±SEM)	Mean weight (g) at fledging (±SEM)	*n*
1	47.95 (±0.37)	205.27 (±3.95)	64
2	49.32 (±0.52)	205.65 (±4.54)	57
3	50.96 (±1.17)	207.21 (±5.98)	24
4	48.00 (±1.00)	226.00 (±56.00)	2
5	47.00	180.00	1

### 3.3 Factors Influencing Nestling Growth Rates

**FIGURE 4:**
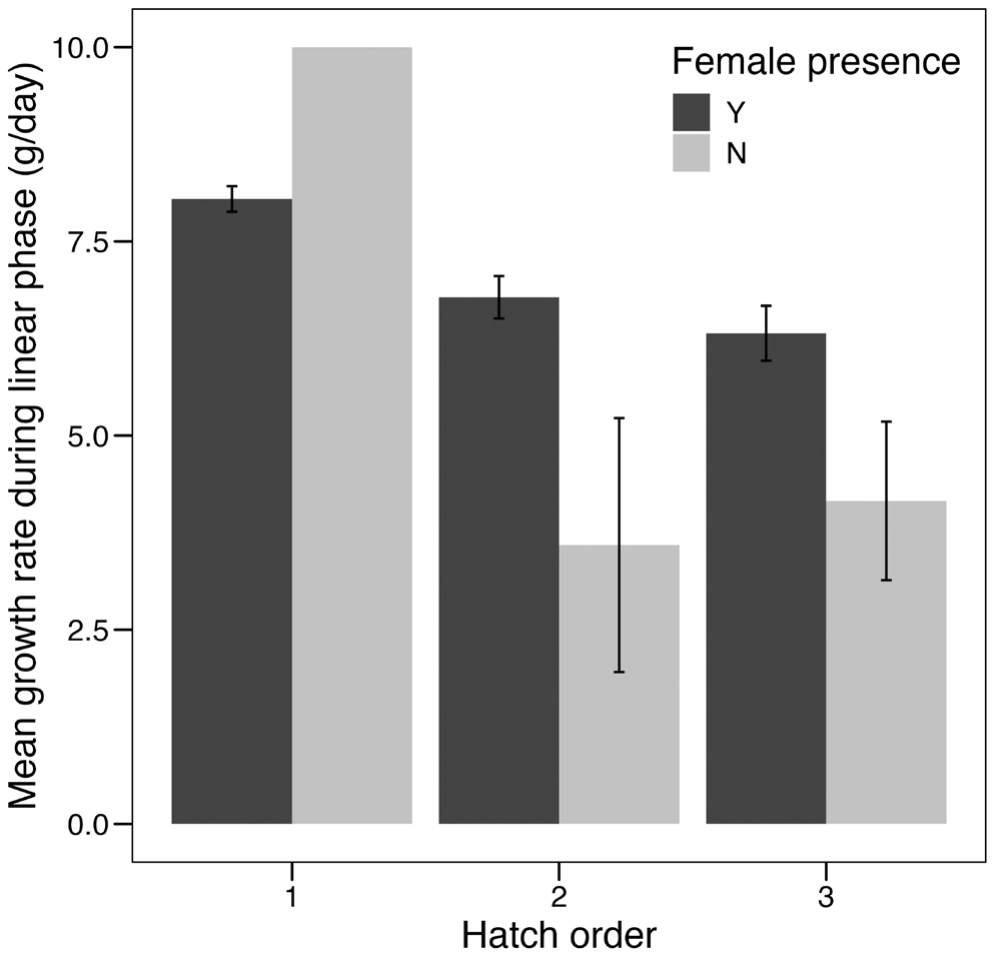
Maternal presence in the nest is positively associated with growth rates of younger nestlings. During the linear phase of growth, later‐hatched nestlings had significantly lower overall growth rates (robust LMM: t = −5.77, df = 111, *p* < 0.001). This difference was mitigated by presence of the female, as later‐hatched nestlings (i.e., hatch order 2 and 3) had relatively higher growth rates when the mother was present in the nest (t = 3.96, df = 19, *p* < 0.001). Error bars represent ±1 SEM. Note that there was only one instance where a female was absent from the nest during the linear growth phase of a first‐hatched nestling, and thus SEM calculation was not possible for that bar.

We then aimed to assess the role of various demographic and environmental factors in shaping individual nestling growth. Based on a linear mixed‐effects model, we found significant effects of hatch order, female presence, and rainfall on rates of nestling growth during the linear phase of growth (Figure 4). Later‐hatched nestlings had significantly lower growth rates than earlier‐born nestlings (robust LMM: *t* = −5.77, df = 111.16, *p* < 0.001), with a decrease of 1.09 (**±**0.19) g/day for every increase in hatch order (e.g., first‐to second‐hatched, second‐ to third‐hatched, etc.; Table 2). Overall, nestlings had higher growth rates when the adult female was present in the nest box (*t* = 3.96, df = 19, *p* < 0.001; Table 2). This pattern was driven by positive effects of maternal presence in later‐hatched nestlings (Figure 4). Greater annual rainfall, a proxy for resource abundance, was also associated with modest but significant increases in overall nestling growth rates (*t* = 3.422.47, df = 85, *p* < 0.001; Table 2).

**TABLE 2:** Output of robust linear mixed‐effects model with growth rate (g/day) during the linear growth phase as a function of hatch order, female presence, and annual rainfall.

	Estimate	SE	df	*t*	*p*
Intercept	6.07	0.70	111	8.62	< 0.0001
Hatch order	−1.09	0.19	111	−5.77	< 0.0001
Female presence	1.90	0.48	19	3.96	< 0.0001
Rainfall (mm)	0.003	0.001	85	3.42	0.001

### 3.4 Factors Influencing Incidence of Brood Reduction

**FIGURE 5:**
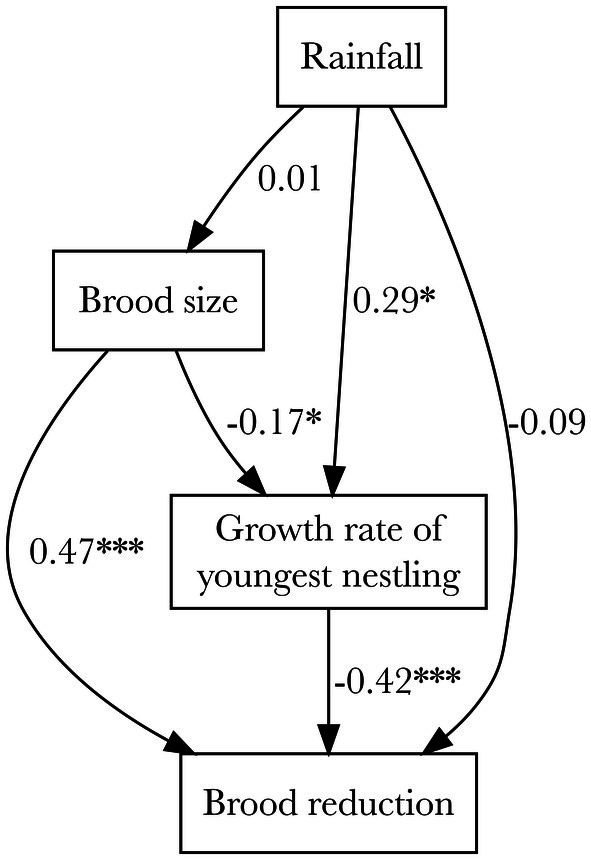
Path model representing causal hypotheses for the direct and indirect mechanisms of brood reduction. Arrows represent the causal direction by which variables could influence brood reduction. Values represent standardized path coefficients, and asterisks denote significant paths (*p < 0.05; **p < 0.01; ***p < 0.001).

We fit a path model outlining our hypothesis for the ways that various causal mechanisms (i.e., resource availability, brood size, and growth rate of the youngest nestling) interact to influence the likelihood of brood reduction occurring in a given nest (Figure 5; Table 3). Based on the model, brood size was a strongly significant predictor of brood reduction (*z* = 7.40, *p* < 0.001), as well as a negative predictor of growth rates of the youngest nestlings for which data were available (*z* = −2.09, *p* = 0.04). The growth rate of the youngest nestling was also a significant direct predictor of brood reduction (*z* = −3.96, *p* < 0.001). We detected no significant direct effects of annual rainfall (a proxy for resource availability) on brood reduction (z = −0.83, *p* = 0.41) or brood size (*z* = 0.07, *p* = 0.95), although annual rainfall did positively predict the growth rate of younger nestlings (*z* = 2.48, *p* = 0.01). When the significance of various indirect and direct effects of rainfall on brood reduction were considered in a mediation model, only the indirect effect mediated by growth rate of the youngest nestling was significant (*z* = −2.00, *p* < 0.05; Table 4).

In sum, brood size and growth rate of younger nestlings strongly predicted incidence of brood reduction, and these results held when an influential outlier point was included in the analysis (Table A1). In contrast, we found no significant direct or total effect of rainfall on brood reduction, although rainfall may indirectly influence the probability of brood reduction via its effects on growth rate of younger nestlings. However, we note that the estimated magnitude of the indirect effect of rainfall on brood reduction was small (Table 4), and the significance of this effect disappeared when an influential outlier point was included in the analysis (Tables A1 and A2).

**TABLE 3:** Output of the path model.

Response variable	Predictor variable	Estimate	SE	Std. path coefficient	*z*	*p*	*R* ^2^
Brood reduction	Brood size	0.50	0.07	0.47	7.40	0.000 ***	0.50
	Growth rate of youngest nestling	−0.20	0.05	−0.42	−3.96	0.000 ***	
	Rainfall	−0.001	0.001	−0.09	−0.83	0.41	
Growth rate of youngest nestling	Rainfall	0.005	0.002	0.29	2.48	0.01 *	0.11
	Brood size	−0.38	0.18	−0.17	−2.09	0.04 *	
Brood size	Rainfall	0.000	0.001	0.01	0.07	0.95	0.00

*Note:* Brood reduction was modeled as a binary response variable (0/1) that could be predicted by growth rate of the youngest nestling (g/day) for which data was available, brood size (# of nestlings in brood), and annual rainfall (mm). Brood size and growth rate of the youngest nestling were modeled as functions of annual rainfall or annual rainfall and brood size, respectively. Asterisks denote significant paths (*p < 0.05; **p < 0.01; ***p < 0.001).

**TABLE 4:** Output of mediation model assessing direct and indirect effects of annual rainfall on brood reduction.

Effects of annual rainfall on brood reduction	Estimate	SE	*z*	*p*
Direct effect	−0.001	0.001	−0.83	0.41
Indirect effect via brood size	0.00	0.00	0.07	0.95
Indirect effect via brood size and growth rate of youngest nestling	0.00	0.00	0.07	0.95
Indirect effect via growth rate of youngest nestling	−0.001	0.00	−2.00	0.045 *
Total effect	−0.002	0.001	−1.60	0.11

*Note:* Potential direct and indirect effects correspond to the various potential connections between annual rainfall and brood reduction in the Figure 5 path model. The only significant effect of rainfall on brood reduction was the indirect effect via growth rate of the youngest nestling.

## 4 Discussion

Brood reduction is a common yet paradoxical phenomenon in birds, and our understanding of its drivers remains incomplete. In this multi‐year study of southern yellow‐billed hornbills ( *Tockus leucomelas* ) in Namibia, we assessed patterns and predictors of nestling growth and brood reduction, considering effects of hatch order, brood size, maternal presence, and the environment. Earlier hatch order, maternal presence, and higher annual rainfall all had significant, positive impacts on nestling growth rates. In addition, brood reduction was more prevalent in larger broods and usually involved the death of the youngest nestling. Because younger nestlings had lower growth rates in brood‐reduced nests, starvation is likely the proximate cause of death in most cases. Finally, our data supported the interpretation that annual rainfall (a proxy for resource availability) may have a weak and indirect influence on the probability of brood reduction via its effects on brood size and growth rates of younger nestlings, although the direct and total effects of rainfall on brood reduction were not significant. Together, these results provide insight into the mechanisms and drivers of brood reduction in hornbills, which may have applications to other *Tockus* species and avian taxa with similar breeding biology (Stander et al. 2021).

Although our data were purely observational, our findings speak to some of the predictions of the brood reduction hypothesis for the function of hatching asynchrony. As predicted by this hypothesis, we observed strong effects of hatch order on nestling growth and mortality. Earlier‐hatched nestlings had significantly higher growth rates throughout the linear and asymptotic phases (Figure 2), and later‐hatched nestlings were more likely to perish than their older siblings (Figure A2). Mortality in later‐hatched nestlings typically occurred in the first few days post‐hatching, before parents had invested considerable resources in the nestling; conversely, cases of mortality in earlier‐hatched nestlings tended to occur at more advanced ages, suggesting that parents try harder to avoid the death of the nestling that represents the greatest sunk cost. Other studies across diverse avian taxa show effects of hatching order on nestling growth rate and mortality, particularly when hatching asynchrony creates large size differences between siblings (Clotfelter et al. 2000; Dey et al. 2014; Hildebrandt and Schaub 2018). Therefore, hatching asynchrony may set the stage for brood reduction in the event of limited resources, a potentially important strategy in semi‐arid habitats where resources can be unpredictable (Howe 1978; Lehmann et al. 2020).

Our data partially supported the prediction that the resource environment influences the likelihood of brood reduction. Although direct and total effects of annual rainfall on brood reduction were not significant, we did observe modest but significant indirect effects of rainfall on brood reduction via its positive effects on growth rates of younger nestlings (Tables 2, 4; Figure 5; but see Tables A1, A2). However, we note that our proxy for resource availability (annual rainfall) was quite coarse, and finer‐grained precipitation and/or resource availability data (e.g., insect surveys, trapping, or measuring provisioning rates) could reveal stronger direct impacts of the environment on brood reduction. Opportunities for these studies will continue to present themselves as the regional climate—and, presumably, resource availability—is likely to become increasingly variable in future years, and path analysis may provide a promising analytical framework for disentangling various direct and indirect mechanisms of brood reduction.

Mothers are known to play a key role in shaping nestling fates during the nesting period, ranging from allocating food among nestlings to actively killing them (Boland et al. 1997; Finnie 2012; José Soler et al. 2022; Soler et al. 2022). In this study, we found that maternal presence in the nest had a significant and positive effect on nestling growth, particularly for later‐born nestlings (Figure 4). This suggests that females may mitigate sibling competition in the nest by ensuring that smaller nestlings are able to obtain their share of the provisions. These results corroborate the findings of Finnie (2012), which demonstrated experimentally that mothers mediate sibling competition in the nest by reducing sibling aggression and increasing the equity with which food is allocated to nestlings. However, the nature of sibling mediation by the female could also depend on resource availability. For example, white‐winged chough ( *Corcorax melanorhamphos* ) parents preferentially feed larger nestlings in natural conditions, but then switch to favoring younger nestlings when given supplemental food (Boland et al. 1997). Future research using nest box cameras to examine female provisioning, nestling begging, inter‐sibling aggression, and male provisioning (Finnie 2012) are needed to better determine the specific mechanisms at play in this population.

Surprisingly, despite substantial mass differences throughout development, we found that nestlings of different hatch orders were similar in mass at the time of fledging (Figure 3A). These results suggest there may not be major fitness consequences of hatch order if later‐hatched nestlings can make it through the crucible of development and survive to fledge. However, we note that we were unable to account for nestling sex when assessing weight at fledging, which may confound our analysis. Similar to our study, previous research in tree swallows ( *Tachycineta bicolor* ) and Eurasian kestrels ( *Falco tinnunculus* ) has shown that hatch‐order effects on nestling mass diminish as nestlings age (Clotfelter et al. 2000; Massemin et al. 2002). However, we cannot rule out the possibility that hatch order impacts fitness after fledging in ways that we did not evaluate in our study. For example, a study in crested ibises (*Nippon nippon*) found that earlier‐hatched individuals enjoy a slew of “silver spoon” effects, including increased adult survival, earlier age at first reproduction, higher lifetime reproductive success, and better offspring body condition (Song et al. 2019). Studies have also explored the impact of hatching order and asynchrony on dominance and mate choice in other avian species (Dey et al. 2014; Mainwaring et al. 2012; Stanback 1994). Whether such long‐term hatching‐order effects would be expected in situations where hornbill offspring fledge with similar mass (and/or body condition) represents an interesting potential area of future research.

Better understanding brood reduction, and overall breeding biology, in African hornbills is useful for species management and conservation, particularly as populations face additional stressors such as extreme weather due to climate change (Keja‐Kaereho and Tjizu 2019; Van De Ven et al. 2019). In addition to changes in prey availability due to a changing climate, southern yellow‐billed hornbills have been shown to experience sharp declines in foraging efficiency and male body condition—presumably affecting the nesting female and nestlings reliant on him—during high temperatures (Van De Ven et al. 2019). Brood reduction, likely facilitated by hatching asynchrony, enables parents to rear some offspring even if resource provisioning is insufficient to rear the entire brood, likely preventing total nest failure in the presence of heat waves. However, the implications of pairs consistently producing lower numbers of fledglings as temperatures increase is unclear. Understanding the impact of higher temperatures on nesting behavior and brood reduction is a critical research area for all African hornbills, and our study serves as a baseline to which future studies can compare breeding changes in *Tockus* species. Southern yellow‐billed hornbill habitat is expected to experience major changes in the coming decades, and better understanding current breeding biology and the brood reduction dynamics will inform conservation efforts.

## 5 Conclusions

As one of the first comprehensive investigations into the predictors of brood reduction in southern yellow‐billed hornbills, this study partially supports the hypothesis that hatching asynchrony may set the stage for brood reduction by enabling mothers to dynamically adjust the number of offspring in a variable resource environment. In line with predictions, hatch order had strong effects on nestling growth and survival, and mothers appear to play a role in mitigating sibling competition while in the nest. Additionally, we found that rainfall (a proxy for resource availability) may have small but significant indirect effects on brood reduction via its effects on growth rates of younger nestlings, although direct and total effects of rainfall on brood reduction were not significant. Future research would benefit from directly assessing resource availability (e.g., via insect surveys) to better understand how the resource environment influences brood reduction dynamics. The adaptive value of hatching asynchrony likely varies between species, but it may serve to facilitate brood reduction in birds with low nest predation in unpredictable resource environments.

## Author Contributions

**Melanie A. Smith:** conceptualization (supporting), data curation (lead), formal analysis (supporting), investigation (equal), methodology (supporting), visualization (supporting), writing – original draft (lead). **H. Luke Anderson:** conceptualization (equal), data curation (equal), formal analysis (lead), investigation (lead), methodology (lead), visualization (lead), writing – review and editing (equal). **Jordan Karubian:** conceptualization (lead), methodology (equal), project administration (lead), supervision (lead), writing – review and editing (equal). **Mark Stanback:** conceptualization (lead), data curation (lead), funding acquisition (lead), investigation (lead), methodology (lead), project administration (lead), supervision (supporting), writing – review and editing (equal).

## Conflicts of Interest

The authors declare no conflicts of interest.

## Data Availability

All data and code files are made available on Dryad, accessible at the following DOI: https://doi.org/10.5061/dryad.jsxksn0pj.

## Appendix A

**TABLE A1:** Output of the path model with outlier point included in analysis.

Response variable	Predictor variable	Estimate	SE	Std. path coefficient	z	p	R^2^
Brood reduction	Brood size	0.51	0.07	0.48	7.32	0.000 ***	0.55
	Growth rate of youngest nestling	−0.21	0.04	−0.50	−4.89	0.000 ***	
	Rainfall	−0.001	0.001	−0.11	−0.92	0.36	
Growth rate of youngest nestling	Rainfall	0.003	0.002	0.14	1.21	0.23	0.03
	Brood size	−0.25	0.18	−0.10	−1.40	0.16	
Brood size	Rainfall	0.000	0.001	−0.01	−0.12	0.90	0.00

*Note:* The same path analysis was conducted as presented in Table 3 and Figure 5, but with an influential outlier point (> 4 SD from the growth rate mean) included in the analysis. Results of this analysis differ from those presented in the main text in that the effects of brood size and annual rainfall on growth rate of youngest nestlings are not significant when the outlier is included. Estimated effects of brood size and youngest nestling growth rate on brood reduction are similar. Asterisks denote significant paths (**p* < 0.05; ***p* < 0.01; ****p* < 0.001).

**TABLE A2:** Direct and indirect effects of annual rainfall on brood reduction, with outlier point included in analysis.

Effects of annual rainfall on brood reduction	Estimate	SE	z	p
Direct effect	−0.001	0.001	−0.92	0.36
Indirect effect via brood size	0.00	0.00	−0.12	0.90
Indirect effect via brood size and growth rate of youngest nestling	0.00	0.00	−0.12	0.91
Indirect effect via growth rate of youngest nestling	−0.001	0.00	−1.15	0.25
Total effect	−0.001	0.001	−1.40	0.16

*Note:* Potential direct and indirect effects correspond to the various potential connections between annual rainfall and brood reduction in the path model. No significant direct, indirect, or total effects of rainfall on brood reduction were detected.
